# Living with a chronic disease: insights from patients with a low socioeconomic status

**DOI:** 10.1186/s12875-021-01578-7

**Published:** 2021-11-18

**Authors:** Lisa Van Wilder, Peter Pype, Fien Mertens, Elke Rammant, Els Clays, Brecht Devleesschauwer, Pauline Boeckxstaens, Delphine De Smedt

**Affiliations:** 1 Department of Public Health and Primary Care, Ghent University, Ghent, Belgium; 2 Department of Human Structure and Repair, Ghent University, Ghent, Belgium; 3 Department of Epidemiology and Public Health, Sciensano, Brussels, Belgium; 4 Department of Translational Physiology, Infectiology and Public Health, Ghent University, Merelbeke, Belgium

**Keywords:** Chronic disease, Health-related quality of life, Socioeconomic status, Health inequalities, Conceptual model

## Abstract

**Background:**

Little is known about how patients with low socioeconomic status (SES) experience their chronic disease, and how it impacts health-related quality of life (HRQoL). Compared to their more affluent counterparts, worse outcomes have been reported. A better understanding of the domains of HRQoL that are relevant to these specific populations is therefore needed. We explored the experiences of living with a chronic disease in low SES persons.

**Methods:**

A qualitative interview study was performed in Flanders, Belgium. Semi-structured interviews were conducted in chronically ill patients, selected through purposive sampling. Interviews were audio-recorded and transcribed verbatim. Analysis followed an inductive and iterative approach.

**Results:**

Fifteen patients were interviewed. Six major themes were identified: a heavy bag to carry, loss of autonomous life, inner and outer loneliness, emotional imbalance, unmet need for support, and coping strategies. Patients experienced their illness as an additional problem on top of all other problems (i.e. financial/social problems, traumatic life events). In general, the disease burden and non-disease burden were mutually reinforcing, resulting in greater dependency, greater risk of social isolation, greater psychological distress, and greater risk of impaired HRQoL.

**Conclusions:**

This study is the first to provide detailed insight into the experiences of living with a chronic disease in low SES persons. A conceptual model is proposed that can be used in daily clinical practice to raise awareness among clinicians and health care providers that the patient’s needs go beyond the disease itself. Future research is needed to validate and test the model.

**Supplementary Information:**

The online version contains supplementary material available at 10.1186/s12875-021-01578-7.

## Introduction

Living with a chronic disease is challenging, as it interferes with physical, mental, and social functions and thus greatly affects a person’s quality of life. Indeed, chronically ill patients are facing major struggles such as higher expenditures, social isolation and loneliness, disabilities, fatigue, pain/discomfort, feelings of distress, anger, hopelessness, frustration, anxiety, and depression [1–5]. In addition to morbidity and mortality data, investigating the patient’s perspective provides a complete overview of the impact of chronic diseases [6]. As a result, the interest into health-related quality of life (HRQoL) in chronic diseases is growing due to a lack of adequate cure and hence the shift from cure to care [7]. HRQoL captures individuals’ self-perceived impact of a medical condition, its symptoms and treatment referring to physical, mental, and social well-being, compared to what they believe to be ideal [7, 8].

While numerous quantitative studies have assessed the impact of chronic diseases on HRQoL in terms of ‘scores’ and ‘values’ [9], a thorough qualitative understanding of how patients experience and cope with their illness is limited and often reflected in vague themes, e.g. ‘emotions of the patient’ [10–13]. Especially, little is known about the experiences of individuals with low socioeconomic status (SES). This particular risk group has been reporting worse outcomes than their more affluent counterparts in terms of morbidity [14], mortality [15, 16], and HRQoL [11]. In addition, coping (defined as cognitive and behavioral efforts to deal with stressful events [17]) resources have also been found to be unequally distributed [18]. Hence, in-depth explorations incorporating individuals’ experiences and viewpoints are needed to better understand HRQoL domains that are relevant to these specific populations.

The aim of this study is to provide a detailed understanding of how individuals with low SES experience their illness and how the illness affects their lives. Face-to-face interviews are used to gain a holistic view on the disease burden in this target group. The results may help us to understand what it is like to live with a chronic disease and its impact on daily life among vulnerable patients, in order to optimize person-centred care and overall HRQoL.

## Methods

### Study design

A qualitative study based on face-to-face semi-structured interviews was performed, following the Consolidated Criteria for Reporting Qualitative Research (COREQ) checklist (Additional file 1) [19].

### Study participants

The study participants were adults (≥ 18 years) who were clinically diagnosed (≥ 6 months) with at least one chronic disease. Educational level, employment status, income and/or financial hardship were used as proxies for low SES [20]. In general, patients who were entitled to supplemented refunds because their income was less than the ceiling amount were considered low SES. Study participants were not eligible if they had major cognitive impairment, insufficient understanding of Dutch, and were unable to give informed consent.

### Recruitment of participants

Purposive sampling was used to obtain a wide variety in patients’ characteristics and hence to ensure a broad range of experiences and thoughts. This technique is widely used in qualitative research in order to identify information-rich cases [21]. Patient recruitment was organized through five general practitioners (GPs) with a practice within five community health centers in Ghent, Belgium (convenience sample [22]), which mainly host patients with lower SES. A community health center is a multidisciplinary practice that offers primary care and aims to reduce the social gradient in health care by removing as many barriers as possible, financially, but also culturally and physically. Each center receives a fixed capita payment for each registered patient, resulting in no co-payment for patients [23]. GPs identified eligible patients during consultation and transferred their contact details to the researcher, with patients’ consent. Recruitment continued until data saturation (i.e. when no new information or additional perspectives are observed in the data when adding more participants [24]) was achieved, which was checked after each interview.

### Data collection

Data were collected through face-to-face semi-structured interviews. After review of the literature, an interview guide (Table 1) with open and semi-open questions was developed in advance and its content was evaluated by highly experienced qualitative researchers. The interviews lasted between 58 and 100 min and were conducted by the principal researcher (LVW) who received training in interview techniques. The interviews took place at the participant’s preferred location, i.e. at the community health center, at the patient’s home, or online (due to COVID-19 pandemic). The interviews were audio-recorded and transcribed verbatim. All interviews were conducted in Dutch and took place between July 2020 and March 2021.

**Table 1:** Interview guide used for semi-structured interviews with chronically ill patients

Topics	Main questions
*Living with chronic disease*	Can you tell me something about your chronic condition? How do you experience your chronic condition? What does your condition mean to you? How does your condition affect your life? What has changed in your life since your diagnosis?
*Physical and mental health*	Do you experience physical limitations because of your condition? How does your condition affect how you feel mentally?
*Social life*	Has your condition affected your social life? How? Has your relationship with others changed because of your condition?
*Coping*	How do you deal with your illness? How do you deal with the limitations as a result of your condition?
*Network and context*	Can you tell me something about the social support you may or may not receive? Does the support meet your expectations? How do you experience the professional care you receive?

### Data analysis

Data were analyzed using the software package NVivo version 12 [25]. A constant comparative method was used within and between interviews with the emphasis on identifying, analyzing, and interpreting patterns by inductively coding the data [26]. The transcripts were open-coded by one researcher (LVW). After analyzing each interview, all codes were compared in order to construct categories and subcategories. The analysis process followed an inductive and iterative approach. The results were higher level themes that form the basis for making statements about patients’ experiences with chronic disease. At regular time intervals, the principal researcher (LVW) and four experts with different backgrounds (DDS, PP, FM, ER) engaged in discussion on the codes, concepts, relationships between concepts, and preliminary results to ensure investigator triangulation (i.e. the participation of two or more researchers in the same study to provide multiple observations and conclusions [27]).

## Results

Out of 15 participants, 8 were male. The mean age was 57.7 years (range 38-83 years). Table 2 summarizes patients’ characteristics. The analysis revealed six major themes: a heavy bag to carry, loss of autonomous life, inner and outer loneliness, emotional imbalance, unmet need for support, and coping strategies (Table 3). The results are presented with illustrative quotes.

**Table 2:** Sociodemographic and disease-related characteristics of the participants

Participant number	Sex	Age	Living situation	Education^**a**^	Employment status	Chronic conditions
P1	M	45	Partner	Medium	Unemployed	Low back pain + recurrent depression
P2	M	45	Single	Medium	Employed (PT)	Cerebral palsy
P3	F	39	Single	Low	Unemployed	Borderline personality disorder + diabetes + knee replacement
P4	M	57	Single	Low	Unemployed	Cardiovascular disease + psychological vulnerability
P5	M	55	Single	Medium	Unemployed	Personality disorder + mild cognitive disorder
P6	M	59	Partner	Low	Unemployed	Chronic pain due to post-polio syndrome
P7	F	83	Single	Low	Retired	Hypertension + arthrosis
P8	M	75	Single	Medium	Retired	Type 2 diabetes + mild cognitive impairment
P9	F	63	Single	Low	Unemployed	Type 2 diabetes + osteoporosis + cataract + incontinence
P10	F	52	Partner	Low	Employed (FT)	Type 2 diabetes
P11	F	38	Single	Medium	Unemployed	Fibromyalgia
P12	F	58	Partner	High	Unemployed	Chronic bronchitis + bipolar personality disorder
P13	M	71	Single	Low	Retired	Hypertension + low back pain + chronic nerve pain
P14	M	72	Partner	Medium	Retired	Parkinson’s disease
P15	F	54	Single	High	Employed (FT)	Recurrent depression

*PT:* part-time, *FT:* full-time

^a^ Classified into low (lower secondary education or less), medium (higher secondary education), and high (higher education)

**Table 3:** Overview of the different themes and subthemes

**Theme 1:** A heavy bag to carry	
Subtheme 1a: Personal life history	
Subtheme 1b: Chronic disease: extra weight and burden	
**Theme 2:** Loss of autonomous life	
Subtheme 2a: Illness dependency	
Subtheme 2b: Dependency on others	
**Theme 3:** Inner and outer loneliness	
Subtheme 3a: Loneliness and social isolation	
Subtheme 3b: Lack of understanding	
Subtheme 3c: Goal lacking	
**Theme 4:** Emotional imbalance	
Subtheme 4a: Negative emotions	
Subtheme 4b: Positive emotions	
**Theme 5:** Unmet need for support	
**Theme 6:** Coping strategies	
Subtheme 6a: Illness acceptance	
Subtheme 6b: Keep on fighting	
Subtheme 6c: Other coping strategies	

### A heavy bag to carry

#### Personal life history

Patients were marked by their life history and living conditions, often related to their lower SES: (childhood) trauma, living in a youth institution, death of loved ones, criminal activity, addictions, harmful relationships, sexual extortion, debts etc. These events strongly impacted their well-being and quality of life, making it extra difficult to deal with challenges such as having a chronic disease. The chronic illness only increased patients’ vulnerability.

*“I may not keep sweets at home, otherwise I’ll eat them immediately. I don’t know why, it’s stronger than myself. Is that a flight? Because of what happened to me in the past? I think if all these things hadn't happened I would have been a slim woman without diabetes. (…) After all these years I still can’t handle the past, it’s there every day. Sometimes I can ignore it, I feel optimistic and start a diet. But that can change the same day. Why am I starving myself? What a stupid cow am I?”* (P10, female, 52 years)

#### Chronic disease: extra weight and burden

The illness was perceived as an additional burden and was experienced differently among patients, e.g. challenging, not easy, burdensome, terrible, hellish, wretched, and disgusting.

*“We see it this way: the backpack is your past that you permanently carry with you and from which you never completely get rid of it. The disease is an extra weight in the bag.”* (P15, female, 54 years)

Patients were constantly reminded of their disease, which they experienced as very confronting, however for some patients the disease became a habit. Patients said it was painful to accept not being able to do things the way they used to, or worse, that they simply cannot do certain things. Some were tortured by unsolvable questions and sometimes had feelings of injustice.

*“You see other people doing things that you can’t do (patient is wheelchair-dependent). During coffee breaks, my colleagues are busy talking about the weekend: what they’ve done with the kids, going out… It’s their right, but it hurts. Then you ask yourself the question: why can’t I do that?”* (P2, male, 45 years)

*“If I no longer felt pain, I would find that strange.”* (P7, female, 83 years)

### Loss of autonomous life

#### Illness dependency

Symptoms (e.g. fatigue, pain, breathlessness, muscle spasms, vertigo) being unpredictable and uncontrollable determined patients’ lives, which gave them feelings of dependency and uncertainty. Activities that felt perfectly fine 1 day can be unmanageable another, forcing patients to live from day-to-day. For some patients, the illness affected the feasibility of future plans and wishes such as family planning, which further provoke the feeling of being controlled by the disease.

*“I need to plan when I want to do something and after that I need to schedule enough rest. But I can’t plan too far in advance because I don't know if my body will allow it. So, I need to plan, but I can't make many plans at the same time. That’s very frustrating. If I go somewhere, I need to know: can I sit there?”* (P11, female, 38 years)

#### Dependency on others

Patients said the hardest part of their life is the loss of autonomy and freedom. In general, they already were dependent on others in their lives (e.g. some were supervised by a judicial administrator to manage their depts), resulting in feelings of hopelessness, powerlessness, and being a burden. Becoming ill only increased these feelings as they needed more assistance to manage daily life (e.g. home help, nurse visits, mobility aid, medications or medical devices).

*“I can’t reconcile myself with the fact that I’m immobile and people have to push me around in a wheelchair. Sidewalk up, sidewalk down. I don’t like to do that. Maybe because I prefer being independent.”* (P14, male, 72 years)

### Inner and outer loneliness

#### Loneliness and social isolation

Most patients experienced deep feelings of loneliness which was mainly related to early life events (e.g. being abandoned as child), and aggravated by becoming ill. Physical restrictions (e.g. pain) hindered patients from participating in social activities. Besides, socializing might even trigger (psychological) fatigue, causing patients to stay home. Hence, they end up in a vicious circle. Patients also felt that others were dropping them because they had to keep saying ‘no’ to social gatherings. Eventually, their limited social network narrowed, resulting in feelings of being left alone, abandoned, and forgotten.

*“People organize things, but you can’t participate. That's lonely. People also need to come to my house: they do that once or twice, but they also want to do an activity with you and… then they drop out and you end up with no visitors. That hurts. That hurts a lot.”* (P3, female, 39 years)

#### Lack of understanding

Loneliness was also related to a lack of support from social interactions, characterized by feelings of being misunderstood and unheard. Patients felt that others were not listening or interested and that they did not understand what the patient was going through.

*“I used to have more friends, but they all dumped me because I couldn't go to town and stuff. First, they said ‘we understand’, but eventually they stopped coming. ‘But you never do anything.’ That hurts. Especially because I mention it in advance, they know it, but they let you down anyway.”* (P3, female, 39 years)

*“When you talk about it with other people, they don't listen. They say: that's not my problem. People only think of themselves, your story doesn't interest them.”* (P4, male, 57 years)

#### Goal lacking

Loneliness was linked to the feeling of boredom because of the loss of activities (e.g. football, drawing, professional activity) due to disease symptoms. Patients felt that they were lacking goals, content, and meaning. The abundance of time to think triggered negative/depressed thoughts and bad mood.

*“I watch a lot of television because I’ve nothing else to do. During the week, I follow several TV series, but there’s nothing on the television during the weekend. And then the days last long.”* (P9, female, 63 years)

*“I no longer had daytime activities, nothing (because of pain). I actually spend a lot of time in bed during the day. In fact, I always lie in my bed when I’m home. I know that's unhealthy.”* (P3, female, 39 years)

### Emotional imbalance

#### Negative emotions

Patients’ emotional state was dominated by negative emotions resulting from past life events (e.g. childhood trauma), current life situations (e.g. being single), and intrapersonal factors (e.g. low self-esteem). The illness only reinforced these negative feelings. Feelings of stress, overthinking, and inner turmoil caused unpredictable/unstable mood and even a depressed mood. The latter was accompanied by feelings of sadness, apathy, and unhappiness. Several patients felt suicidal in the past.

*“I’ve already had suicide attempts. Somehow, I always get through it. However, the dark thoughts will remain. I’m convinced that there will always be something on my path that will pull me down again.”* (P15, female, 54 years)

Some patients felt guilty for having the illness (although they knew it was out of their control), for cancelling plans, calling out of work, being a ‘burden’ to others etc.

*“I feel like a weakling because I’ve ended up in a depression again. Then I’m not satisfied with myself. It harms my self-confidence. I’m still wondering: is it my own fault?. I don't know, I don't know where depression comes from. (…) I’ll probably continue to feel guilty about being absent from work in the future.”* (P15, female, 54 years)

Patients experienced illness-induced shame which arose with an unexpected loss of control over bodily functions. As a result, they often receive compassion and patronizing, which reinforced feelings of being burdensome and miserable. One patient was ashamed of her depression and kept it a secret because having mental illness is taboo.

*“Wherever I am, I’m always the only one who coughs. I find that very annoying because people are watching, especially now with COVID-19. I’m actually ashamed. That’s not a nice feeling. I feel uncomfortable, I would rather not be there.”* (P12, female, 58 years)

Patients were concerned about how their situation would evolve, knowing that the disease is progressing, that they are getting older, and that they will need even more help. Their limited financial and social resources reinforced these future-oriented anxieties. Other patients feared losing autonomy, losing others, being rejected, misunderstanding from others, and death.

*“ I’m only 39 years old and I already have so many problems, what when I turn 60? I don’t have money for additional care. And who will help me? I have nobody. I do worry about that.”* (P3, female, 39 years)

#### Positive emotions

In case patients had a partner/family/children/colleagues, they were thankful for their help and support. Patients appreciated that they could always count on their GP to discuss confidential issues. Some patients visited an organization for people with psychological vulnerability who have difficulty connecting with society and were grateful for it as it is their only source of social contact and because it gave them a sense of belonging and companionship. Talking to someone, even small talk, is experienced as a relief. Patients also expressed being satisfied and thankful for the (sometimes) little they can and have. Few patients were satisfied with almost everything in their life. One patient felt the illness expanded his world and made him grow as a person. Some patients felt proud of what they have already overcome in life.

*“I’m satisfied. With my television, my bed, my washing machine. That's it for me. I don't care about the rest. It's that simple. Having no worries, just the little things.”* (P4, male, 57 years)

*“I’m happy with who I am. I am who I am and that's quite okay.”* (P11, female, 38 years)

### Unmet need for support

Patients wanted more persons in their life to get support from, especially for emotional support, although they do not want to bother other people. Some felt they had nobody to count on – they could not call anyone if they need help or if they just want to have a chat.

*“I sometimes call the outreach worker or my financial administrator. Then I make up an excuse or I say: I’m just calling to say hello. Well, I do that just to hear someone, to hear a voice.”* (P3, female, 39 years)

Most patients experienced a lack of financial comfort and support as a result of antecedents (e.g. debts) or SES. The diagnosis of disease made them even more economically vulnerable (e.g. disabilities caused job loss), with sometimes not enough money left for additional care. Some had to choose between living necessities and essential medical care or they had to postpone it, as the allowance could not afford both.

“*I actually need a lot more help. But I just don’t have the money for it.”* (P3, female, 39 years)

*“My teeth have deteriorated a lot since I became ill. I actually postpone the care for as long as possible because that will really cost a lot.”* (P11, female, 38 years)

*“The price of tobacco has increased again, but my spending money remains the same. If you then receive the weekly allowance, you sometimes have to make choices.”* (P3, female, 39 years)

One patient talked about the need for additional disease-related support such as stronger medications or the ability to consult health professionals outside the health community center.

*“I used to have a good physiotherapist. I can go to the physiotherapist outside the community health centre, but then I have to pay for it all by myself.”* (P2, male, 45 years)

### Coping strategies

#### Illness acceptance

Patients believed that they first had to accept the disease in order to cope and to continue their lives. One patient said she will never accept her depression – she keeps fighting to conquer it.

*“All I can say is: try to accept. Only then you can do something about it.”* (P6, male, 59 years)

*“I don't see myself as the disease. I am X with fibromyalgia. And that’s bad luck. But that's okay.”* (P11, female, 38 years)

#### Keep on fighting

No matter how difficult patients experienced their disease and life in general, they stressed the importance of perseverance – “keep fighting and never lose the spirit”.

*“I don’t give up, I don’t give up. I will never give up. No. I’ve been through all those things in my life and yet, I keep on fighting.”* (P1, male, 45 years)

In difficult moments, it was important to pull themselves together as quickly as possible and to stay strong, especially for the (few) people around them. Some patients were stubborn – they did everything they could to not be inferior to others. Asking for help was seen as a final solution. In case patients got desperate (e.g. when in pain), they searched for opportunities (e.g. pain clinic) in order to give themselves hope and perspective.

#### Other coping strategies

Patients had to learn to live with the disease requiring major adjustments. A subset developed resilience and successfully integrated it into their lives. Patients had to balance every activity against their physical/mental capacities by listening to their body. One patient considered this as an advantage – the illness forced her to live more slowly and consciously. Patients needed to schedule sufficient days of rest, for both their physical and mental health. However, patients found these activities worthwhile, even if they knew they would suffer from it afterwards.

*“Listening too much to the body can lead to fear of movement and keeps you from doing things, even if they are feasible. I just have to adapt to what is achievable for myself and take that into account. And sometimes I just ignore the pain. Or when I catch up with a friend, I'm like: I'm not sick today. Then I put the disease aside and know I'll be flat the day after. So I think carefully: for what purpose do I want to have extra pain and lie down for a day?* (P11, female, 38 years)

Positive distraction, by planning simple activities (e.g. photography, walking, listening to music, domestic tasks), was perceived as very useful to clear their mind away from the disease/pain or negative emotions. Other patients resorted to substance (a)buse, something they already did before the illness, which offered them consolation.

*“If you keep yourself busy, you will feel the pain less. It pushes the pain to the background. I think it's better than taking a painkiller.* (P6, male, 59 years)

*“Alcohol was my escape.”* (P5, male, 55 years)

Patients engaged in social avoidance behaviours such as not talking to others about their problems or isolating themselves from the outer-world. However, another patient recommended talking openly to others, especially in case of mental illness.

*“The best remedy for me is not to talk about it. Talking about it only brings it back to my mind.”* (P3, female, 39 years)

*“What do I have to say? I have enough problems myself, I’m not going to project my problems onto other people.”* (P14, male, 72 years)

Complaining and cursing brought relief in the moment itself, however, in retrospect, patients admitted that it did not help them move forward.

*If I talk and whine about it every day, it won't go away, will it? So why would you complain about it?* (P7, female, 83 years)

Patients emphasized the importance of putting things in perspective: comparing themselves to others who are worse off (hence realizing they can still do many things), using humour, pulling themselves up to the small things in life.

*“Sometimes I laugh that I’m fat. I believe that helps, to laugh off the problem.”* (P3, female, 39 years)

Some patients said it was important to prioritize yourself and to allow help, however, sometimes patients are too proud.

*“If it doesn't go well, so be it. That’s something I’ve learned. That stubbornness has disappeared a bit.”* (P6, male, 59 years)

The following coping strategies were also perceived as helpful: religious faith (praying does not take the pain away, it only gives strength/courage/hope to endure pain), talking to fellow-sufferers, and having a pet (provides companionship and friendship).

### Conceptual model

**Fig. 1:**
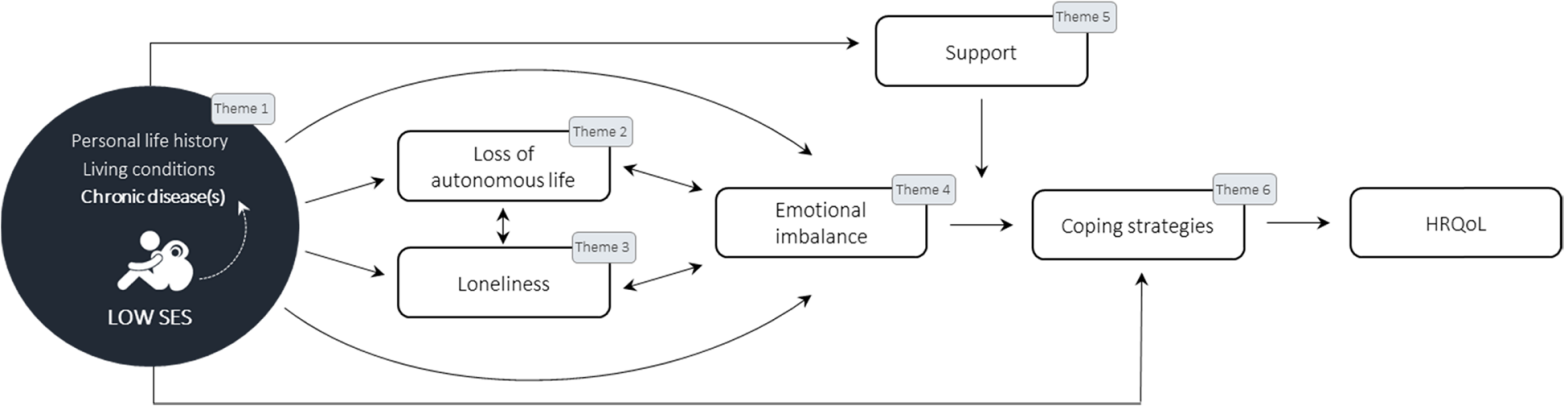
Conceptual model of the impact of chronic disease in individuals with low socioeconomic status (SES)

Based on our findings, a conceptual model (Fig. 1) was proposed reflecting the six core concepts most relevant to the HRQoL construct in chronically ill patients with low SES. The model emphasizes the interaction between concepts and their (in)direct influence on patients’ HRQoL. The hypothesized dynamics suggest that individuals are continuously confronted with stressors that trigger psychosocial reactions that, in turn, negatively affect coping behaviour and subsequently HRQoL. Explanations for the relationships between the concepts are discussed in detail below.

## Discussion

To our knowledge, this is the first qualitative study exploring how people with low SES experience their chronic disease. The findings of the in-depth exploration were translated into a generic conceptual model. Although several conceptual models have already been published [28–31], previous studies primarily focused on middle- to upper-class patients while this study extensively investigated the perspective of socioeconomically disadvantaged groups. Our findings are particularly useful for health care professionals who monitor chronically ill patients. It can guide them in optimizing treatment, recognizing unmet practical and emotional needs, enabling patients to cope with their illness, and thus helping them to live best possible life [13].

Low SES persons are typically faced with a complexity of stressors: high levels of financial and social problems, job insecurity, poor-quality housing, traumatic events etc. [32] The illness was perceived as an additional problem on top of all other problems, reinforcing the experienced burden (e.g. greater care-dependency, greater risk of social isolation and loss of work) - often referred to as ‘cumulative disadvantage’ [33]. These stressors are hypothesized to directly influence the concepts of autonomy, loneliness, emotional well-being, support, and coping. Most of these relationships have been confirmed by the literature [34–43].

Literature also confirms that chronic diseases involve loss of independence [38, 44]. Patients cannot live the life they lived before or the life they imagined living [38], often referred to as ‘chronic sorrow’ which reflects long-term periodic sadness in reaction to continuous personal and social losses that are part of chronic illness or disability [45, 46]. Previous research demonstrated how dependency increases negative emotions as well as reducing self-efficacy, which is important in order to cope properly [47].

Our results furthermore emphasize that loneliness and social isolation are distinct concepts: some patients felt lonely despite having frequent social contacts, while other patients with infrequent social contact did not feel lonely [35]. As such, ‘loneliness’ can be defined as the subjective feeling of being isolated and ‘social isolation’ as the objective state of having few social relationships [35, 48]. More importantly, both concepts are found to have a direct negative impact on one’s mental well-being and thus on the overall quality of life [49, 50].

The above described factors (low SES, being chronically ill, loss of dependence, loneliness) are associated with negative emotions [47, 51–53]. More specifically, chronically ill patients are more susceptible to negative psychological states as a result of changes associated with the disease [54, 55]. In addition, emotional inequalities exist: the lower one’s socioeconomic position, the more likely negative emotions are experienced [56, 57]. Literature showed that SES affects coping strategies by influencing psychological well-being [58, 59]. As in our study, patients’ lower psychological well-being is hypothesized to negatively affect coping behavior, which in turn lowers HRQoL.

Patients experiencing a lack of social, financial, and health-related support negatively influenced the relationship between emotional well-being and coping strategies. Literature hypothesized the mediating role of social support in the relationship between psychological well-being and health-risk behaviors (e.g. smoking), but not specifically in coping [60].

Finally, previous research confirmed coping resources are socially patterned [61]: individuals with lower SES have scant resources (e.g. small social network, little money), resulting in lower sense of personal control or mastery and a limited tendency to directly tackle problems during stressful encounters [18, 62]. The effect of SES on coping behavior would be mediated by control beliefs: low SES individuals have lesser believe in life’s controllability leading them to engage in less efficacious coping strategies that may exacerbate their problems even more [59]. It is therefore not surprising that a maladaptive coping style (e.g. avoidance behavior, self-blame, negation, social withdrawal) was often reported. Previous findings indicate that high SES is positively associated with adaptive coping (e.g. acceptance, positive reappraisal, seeking for social support) which is associated with higher adaptation, well-being, and quality of life while low SES is positively associated with maladaptive coping, which is related to poorer health outcomes [63, 64].

### Implications for research and/or practice

Findings suggest that patients’ needs should not be reduced to disease-related needs only - focusing on the bigger picture is more important. It is crucial for health care professionals to acknowledge the role of social factors (e.g. by providing patients with a social prescription, connecting them to other services [65]), to pay attention to signs of, for instance loneliness, and to create a safe environment where patients feel at ease to share concerns, needs, and feelings. Furthermore, more cohesive support within the health care system should be provided. For example, it is important to be fully informed about the personal circumstances of the patient. In this way, treatment, follow-up, and communication can be tailored to the personal and social characteristics of the individual patient, as reflected in person-centered care [66]. Moreover, patients should be more assisted to strengthen their resources in order to cope with the disease and other stressful life events – which is extra challenging in this target group. It is however important to note that individual coping cannot overcome social disadvantage such as poverty. An important resource is resilience, defined as the ability to cope with traumatic and stressful events and to overcome them in an effective and positive way [64, 67]. Strengthening patients’ resilience would result in adaptive coping through better control of the patient’s behavior and emotions and mental flexibility, and appropriate interaction with the social environment. At the same time, this relates to positive outcomes in terms of HRQoL. To further improve HRQoL in patients with low SES, an individual approach is recommended. Indeed, a “one size fits all” approach might not work because low SES persons differ in many aspects (i.e. heterogeneity in skills, life history, support) and thus different needs and target for intervention. Only by considering individual preferences and priorities, HRQoL can be improved.

### Strengths and limitations

An important strength is the development of a generic conceptual model that fit the realities in clinical practice of patients with complex health and social needs, often neglected by disease-specific pathways [68]. After 12 interviews, no additional topics emerged from the data indicating data saturation was reached. However, we conducted three additional interviews to increase the credibility of the results. In addition, Lincoln and Guba’s criteria were used to check the trustworthiness and rigour of the qualitative data [69].

A number of limitations should be acknowledged. First, the sample consisted of different ages and gender, however, diversity in cultural background was not achieved. Participants in this study were Caucasian who spoke the language of interest. As a result, our results could not be transferred to people of other ethnic backgrounds. Second, we cannot conclude whether our findings are unique to the population we studied. Hence, an interview study with high SES patients is recommended to obtain insights into the intersectionality of the different social classes. Third, the causal sequence of findings visualized in the conceptual framework is based on hypothesis. Hence, quantitative research is needed to test the model.

## Conclusion

In conclusion, this study is the first to provide detailed insight into the experiences of living with a chronic disease in patients of lower SES. The results were incorporated into a newly proposed conceptual model and can be used in clinical practice and during education to raise awareness among clinicians and health care providers that the patient’s needs go beyond the disease itself. Future research is needed to validate and test the model.

## Supplementary Information

**Additional file 1.** COREQ (COnsolidated criteria for REporting Qualitative research) Checklist.

## Data Availability

To protect the anonymity of participants, the qualitative data used in this research cannot be made publically available. Data may be made available upon reasonable request from the corresponding author.

## Abbreviations

HRQoL: Health-related quality of life
SES: Socioeconomic status
COREQ: Consolidated Criteria for Reporting Qualitative Research
GP: General Practitioner
